# *NETO2* Is Deregulated in Breast, Prostate, and Colorectal Cancer and Participates in Cellular Signaling

**DOI:** 10.3389/fgene.2020.594933

**Published:** 2020-12-10

**Authors:** Maria S. Fedorova, Anastasiya V. Snezhkina, Anastasiya V. Lipatova, Vladislav S. Pavlov, Anastasiya A. Kobelyatskaya, Zulfiya G. Guvatova, Elena A. Pudova, Maria V. Savvateeva, Irina A. Ishina, Tatiana B. Demidova, Nadezhda N. Volchenko, Dmitry Y. Trofimov, Gennady T. Sukhikh, George S. Krasnov, Anna V. Kudryavtseva

**Affiliations:** ^1^Center for Precision Genome Editing and Genetic Technologies for Biomedicine, Engelhardt Institute of Molecular Biology, Russian Academy of Sciences, Moscow, Russia; ^2^A. N. Severtsov Institute of Ecology and Evolution, Russian Academy of Sciences, Moscow, Russia; ^3^National Medical Research Radiological Center, Ministry of Health of the Russian Federation, Moscow, Russia; ^4^National Medical Research Center for Obstetrics, Gynecology and Perinatology Named After Academician V.I. Kulakov, Ministry of Health of the Russian Federation, Moscow, Russia

**Keywords:** *NETO2*, breast cancer, prostate cancer, colorectal cancer, fish model

## Abstract

The *NETO2* gene (neuropilin and tolloid-like 2) encodes a protein that acts as an accessory subunit of kainate receptors and is predominantly expressed in the brain. Upregulation of *NETO2* has been observed in several tumors; however, its role in tumorigenesis remains unclear. In this study, we investigated *NETO2* expression in breast, prostate, and colorectal cancer using quantitative PCR (qPCR), as well as the effect of shRNA-mediated *NETO2* silencing on transcriptome changes in colorectal cancer cells. In the investigated tumors, we observed both increased and decreased *NETO2* mRNA levels, presenting no correlation with the main clinicopathological characteristics. In HCT116 cells, *NETO2* knockdown resulted in the differential expression of 17 genes and 2 long non-coding RNAs (lncRNAs), associated with the upregulation of circadian rhythm and downregulation of several cancer-associated pathways, including Wnt, transforming growth factor (TGF)-β, Janus kinase (JAK)-signal transducer and activator of transcription (STAT), mitogen-activated protein kinase (MAPK), and phosphatidylinositol 3-kinase (PI3K)/protein kinase B (AKT) pathways. Furthermore, we demonstrated the possibility to utilize a novel model organism, short-lived fish *Nothobranchius furzeri*, for evaluating *NETO2* functions. The ortholog *neto2b* in *N. furzeri* demonstrated a high similarity in nucleotide and amino acid sequences with human *NETO2*, as well as was characterized by stable expression in various fish tissues. Collectively, our findings demonstrate the deregulation of *NETO2* in the breast, prostate, and colorectal cancer and its participation in the tumor development primarily through cellular signaling.

## Introduction

*NETO2* (neuropilin and tolloid-like 2) was first described in 2001 as a gene demonstrating significant similarity to *NETO1* (Stohr et al., 2002). Both genes encode putative transmembrane proteins containing two CUB domains, followed by a low-density lipoprotein class A (LDLa) module in the extracellular region and conserved FXNPXY-like motif in the cytoplasmic region. This structure of NETO proteins suggested their possible role in intracellular signaling pathways. Furthermore, a series of reports have shown the expression of *NETO2* in the brain, as well as its involvement in the modulation of most kainate receptors (KARs) (Straub et al., 2011) and N-methyl-D-aspartate (NMDA) receptors (Wyeth et al., 2014). Additionally, the interaction of NETO2 with other neuronal proteins, including glutamate receptor-interacting protein (GRIP) (Tang et al., 2012) and K(+)-Cl(−) cotransporter (KKC2), has been reported (Ivakine et al., 2013). Currently, NETO2 and its paralog NETO1 are widely recognized as the main auxiliary subunits of KARs.

More recently, *NETO2* was shown to be involved in carcinogenesis. Increased expression of the *NETO2* gene has been observed in proliferating infantile hemangiomas (Calicchio et al., 2009), hepatocellular (Villa et al., 2016) and nasopharyngeal carcinomas (He et al., 2019), as well as in renal (Snezhkina et al., 2018), lung (Oparina et al., 2012), colorectal (Hu et al., 2015; Fedorova et al., 2017), gastric (Liu et al., 2019), and pancreatic (Li et al., 2019) cancers. Hu et al. (2015) have revealed that *NETO2* overexpression significantly correlates with advanced tumor stage, invasion, and metastasis, as well as increases the risk of patient death in colorectal cancer. In patients with gastric and pancreatic cancers, a similar correlation was observed between *NETO2* expression and tumor progression and worse overall survival (Li et al., 2019; Liu et al., 2019). Reportedly, *NETO2* is related to five-gene transcriptomic signatures predicting rapidly growing tumors and survival in patients with hepatocellular carcinoma (Villa et al., 2016). Furthermore, *NETO2* has been observed in a macrophage-related gene signature predicting resistance to targeted therapeutics, as well as survival, in glioma patients (Sun et al., 2019). Additionally, *NETO2* expression is reportedly associated with the deletion of the *PTEN* (Phosphatase and tensin homolog) gene and high intratumor heterogeneity in prostate cancer (Yun et al., 2019).

In this study, we analyzed *NETO2* expression in prostate, breast, and colorectal cancer samples obtained from Russian patients. In these sample sets, both increased and decreased *NETO2* expression were observed, with no significant correlation between its mRNA levels and primary clinicopathological tumor characteristics. Additionally, we performed shRNA-mediated *NETO2* knockdown in the colorectal cancer cell line, HCT116, resulting in the differential expression of 17 genes and 2 long non-coding RNAs (lncRNAs), as well as deregulation of several cellular pathways, including tumor-associated Wnt, transforming growth factor (TGF)-β, Janus kinase (JAK)-signal transducer and activator of transcription (STAT), mitogen-activated protein kinase (MAPK), and phosphatidylinositol 3-kinase (PI3K)/protein kinase B (AKT) pathways. Moreover, we revealed a high similarity between human *NETO2* and its ortholog in fish *Nothobranchius furzeri*, which demonstrate the shortest captive lifespan for a vertebrate (3 months), a novel model for investigating aging and aging-related pathologies, including cancer (Terzibasi et al., 2007). Additionally, we evaluated gene expression in different tissues of *N. furzeri* using quantitative PCR (qPCR). These results can help to generate unique fishes with gene overexpression/downregulation in the target tissues to establish its role in tumorigenesis [for example, using CRISPR activation (CRISPRa) or interference (CRISPRi) tools].

## Materials and Methods

### Patients and Tumor Samples

In total, 74 colorectal, 40 prostate and 32 breast tumors, with paired adjacent normal tissues, were collected at the National Medical Research Radiological Center, Ministry of Health of the Russian Federation. No patient received chemotherapy, radiation, targeted therapy, and/or immunotherapy before surgery. Postoperative tumor and normal tissues were immediately frozen in liquid nitrogen and store at −80°C. This study was approved by the Ethics Committee of P.A. Hertsen Moscow Cancer Research Institute, Ministry of Health of the Russian Federation. All experiments were performed in strict accordance with the principles outlined in the Declaration of Helsinki (1964). The patients provided written informed consent to participate in this study. The clinicopathological characteristics of the patients and tumors are presented in Supplementary Tables S1–S3. Additionally, colorectal cancer samples were genetically characterized by mutations in *KRAS*, *NRAS*, and *BRAF*, as well as the microsatellite instability (MSI) status. Analysis of mutations in the “hot spots” of the *KRAS*, *NRAS*, and *BRAF* genes was performed in Evrogen (Russia) using the qPCR with following validation of the results with Sanger sequencing. The MSI detection was carried out in the same company using PCR amplification of microsatellite markers (*BAT25*, *BAT26*, *D2S123*, *D5S346*, and *D17S250*).

### RNA Isolation and cDNA Synthesis

Tumor and normal tissues were homogenized in a lysis buffer using the MagNA Lyser Instrument (Roche, Switzerland). RNA was isolated from these tissues, as well as from cell cultures, using MagNA Pure Compact RNA Isolation Kit (Roche) on a MagNA Pure Compact System (Roche). The isolated RNA was quantified using a fluorometer, Qubit 2.0 (Thermo Fisher Scientific, United States). RNA quality was measured on an Agilent Bioanalyzer 2100 (Agilent Technologies, United States). Reverse transcription was performed from 1 mg of RNA using Mint Reagent Kit (Evrogen, Russia).

### qPCR

Quantitative PCR was performed using TaqMan Gene Expression Assay (Thermo Fisher Scientific), primers and probes, for the *NETO2* gene (Hs00983152_m1). *GAPDH*, *GUSB*, and *B2M* were used as reference genes for prostate, colorectal, and breast cancer samples, respectively. Primers and probes for reference genes are shown in Supplementary Table S4. qPCR was performed on an Applied Biosystems 7500 Real-Time PCR System (Thermo Fisher Scientific) according to the scheme described in Krasnov et al. (2015). Each reaction was performed in triplicate. Data obtained by qPCR were analyzed using the ddCt method and the original ATG software (Kudryavtseva et al., 2018). *NETO2* expression was considered meaningful if at least a 2-fold expression was determined owing to potential variations in the reference gene expression.

### Cell Culture

The human colorectal cancer HCT116 cell line was cultured in DMEM (Dulbecco’s Modified Eagle’s medium; PanEco, Russia) supplemented with 10% FBS (fetal bovine serum; Thermo Fisher Scientific) and 100 units of penicillin/streptomycin (Thermo Fisher Scientific) in a humidified incubator at 37°C and 5% CO_2_.

### Cell Transfection

shRNA targeting the nucleotide residues 509–529 of the human *NETO2* gene (NCBI Gene ID: 81831, location 16q12.1) protein-coding region was designed and synthesized as follows: Sense, 5′-GATCCGCGCCAAATTATCCTGACTCATCACGTGATGAG TCAGGATAATTTGGCGTTTTTG-3′, and antisense, 5′-AATTC AAAAACGCCAAATTATCCTGACTCATCACGTGATGAGTC AGGATAATTTGGCGCG-3′. This pair of oligonucleotides and non-specific control scrambled (SCR) shRNA were cloned into BamH and EcoR sites of pLSLP plasmid to establish the pLSLP-anti-NETO2 lentiviral vector. Lentiviral constructs were transfected into 293T cells using Lipofectamine LTX reagent (Thermo Fisher Scientific). Virus-containing supernatants were collected 24 h after the transfection and were used to infect target HCT116 cells in triple repeats with the addition of 5 μg/mL polybrene (Sigma, United States). Infected cells were selected using the regular growth medium containing 1 μg/mL puromycin (Sigma).

### Western Blot Analysis

Western blot analysis was performed as previously described (Kudryavtseva et al., 2016). For *NETO2* detection, the primary recombinant antibody EPR3497 (Abcam, United States) was used.

### Transcriptome Sequencing and Analysis

For cDNA library preparation, we utilized RNA isolated from cell cultures with RIN (RNA integrity number) values ≥8. Libraries were prepared with TruSeq Stranded mRNA Library Prep Kit (Illumina, United States) according to the manufacturer’s recommendations. Transcriptome sequencing was performed on a NextSeq 500 System (Illumina) using 76 base pair single-end reads. At least 30M reads were obtained for each sample. The primary analysis of raw sequences was performed as previously described (Pudova et al., 2019). Then, data as transcripts per million (TPM) were imported in the R environment. Differential gene expression was analyzed using the DESeq2 package of Love et al. (2014). The Kyoto Encyclopedia of Genes and Genomes (KEGG) database was used for pathway enrichment analysis.

### Analysis of Orthologs

Nucleotide and amino acid sequences of *NETO2* in human samples and fishes were aligned using Blastn and Blastp, respectively. Gene expression data were obtained from the Bgee database^1^.

### *Neto2b* Expression Analysis in *N. furzeri*

The eggs of fish *N. furzeri* GRZ were obtained from a commercial supplier and were bred in the Aquatic Housing System (Aquaneering, United States) at the Center for Precision Genome Editing and Genetic Technologies for Biomedicine at the Engelhardt Institute of Molecular Biology. The study was approved by the Ethics Committee of the A.N. Severtsov Institute of Ecology and Evolution Russian Academy of Sciences (approval no. 27, 9.10.2019).

Eight tissue samples (brain, intestines, heart, head kidney, liver, stomach, muscles, and skin) from a female *N. furzeri* were subjected to RNA isolation using QIAzol lysis reagent (Qiagen, Germany), subsequently treated with DNase I (Thermo Fisher Scientific). Quantification of the isolated RNA was performed using the NanoDrop 1000 spectrophotometer (Thermo Fisher Scientific). Reverse transcription was carried out with 500 ng of RNA using RevertAid First Strand cDNA Synthesis Kit (Thermo Fisher Scientific). A pair of primers (forward: CCACCCAACAAGGAGTGTGT and reverse: CCCGTGGAGGTAACAAGACC) was designed for *neto2b* gene detection by qPCR, performed on the Rotor-Gene Q 5 plex HRM (Qiagen) using the following scheme: 95°C for 10 min, 40 cycles of 95°C for 15 s, and 62°C for 60 s.

### Statistical Analysis

The significance of differences observed between two groups (tumors/normal tissues and control cells/treated cells) was assessed using the non-parametric Mann-Whitney *U* test. Correlation analysis was performed using Spearman’s rank correlation coefficient (*r*_*s*_). For transcriptome analysis, the Benjamini-Hochberg method was used to calculate the adjusted *p*-values [the false discovery rate (FDR)]. Differences and correlations were considered significant at a *p*-value of < 0.05.

## Results

### *NETO2* Expression in Breast, Prostate, and Colorectal Cancer

**FIGURE 1:**
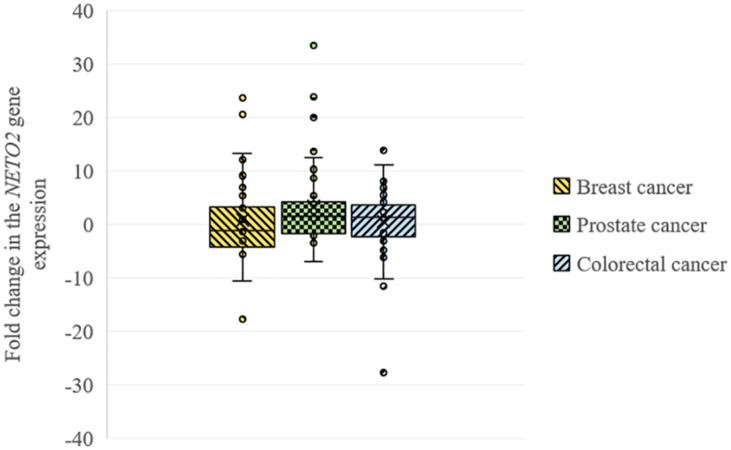
Box plots for *NETO2* expression changes in breast, prostate, and colorectal cancer.

Using qPCR, we analyzed the relative *NETO2* mRNA level in sets of breast (*n* = 32), prostate (*n* = 40), and colorectal (*n* = 74) cancer samples. In breast cancer, *NETO2* gene expression was increased in 31% of cases (2–24-fold), with decreased mRNA levels detected in 44% of samples (2–18-fold) (Table 1). *NETO2* expression was characterized by a 2–33-fold average increase in 40% of prostate cancer samples and a 2–7-fold average decrease in 22.5% of cases. In colorectal cancer, *NETO2* mRNA levels were increased in 35% (2–14-fold) of investigated samples and decreased in 26% of cases (2–28-fold). The highest median value of altered gene expression was determined in prostate cancer (Table 1). In general, a similar trend of *NETO2* expression was observed in investigated cancer types (Figure 1).

**TABLE 1:** Frequency of mRNA level changes in breast, prostate, and colorectal cancer.

**Tumor**	**Frequency of mRNA**		**Median of mRNA**
	level changes, % (n)*		**level changes, n-fold**
	**Increase**	**Decrease**	
Breast cancer	31 (10/32)	44 (14/32)	1
Prostate cancer	40 (16/40)	22.5 (9/40)	1.8
Colorectal cancer	35 (26/74)	26 (19/74)	1.2

*p-value <0.05.

Furthermore, we performed a correlation analysis between several clinicopathological characteristics of tumors (stage for all cancers, differentiation for prostate and colorectal cancer, mutation, and MSI status for colorectal cancer) and *NETO2* expression. The highest correlation coefficients were observed between *NETO2* expression and pathological stage for breast cancer (*r_*s*_* = 0.15) and prostate cancer (*r_*s*_* = 0.25), tumor differentiation for prostate cancer (*r_*s*_* = −0.16), and *BRAF* mutation status (*r_*s*_* = 0.18) for colorectal cancer. Correlation coefficients of less than 0.1 were observed between the NETO2 mRNA level and other investigated clinicopathological characteristics.

### Stable Knockdown of *NETO2* in HCT116

**FIGURE 2:**
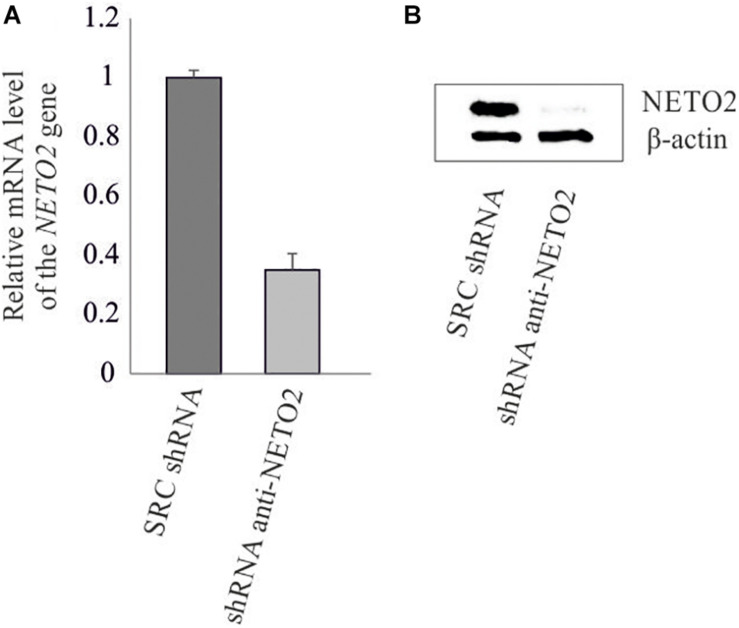
shRNA-mediated knockdown of *NETO2* in the HCT116 cell line. Relative *NETO2* expression in HCT116 cells transfected with targeting shRNA and non-specific SCR shRNA was measured using qPCR at mRNA level **(A)** and western blotting **(B)** at the protein level.

The pLSLP lentiviral vector expressing shRNA targeting *NETO2* (pLSLP-anti-NETO2) and the vector expressing non-targeting SCR shRNA were transfected into HCT116 in triplicate. Protein and mRNA levels of the *NETO2* gene were measured in experimental and control HCT116 cells using western blotting and qPCR (Figure 2). The results obtained confirmed the *NETO2* knockdown.

### Effects of *NETO2* Knockdown on Gene Expression

**FIGURE 3:**
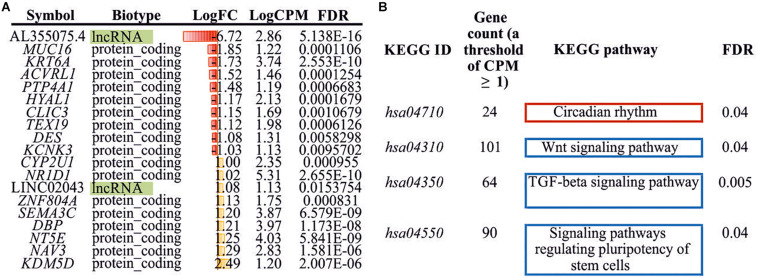
Differently expressed genes/lncRNAs and enriched KEGG pathways. **(A)** DEGs/DE lncRNAs (Log2FC ≥ 1, Log2FC ≤ −1, CPM ≥ 1, FDR < 0.05) identified between experimental and control HCT116 cells. **(B)** Enriched KEGG pathways (FDR < 0.05) with DEGs (CPM ≥ 1, FDR < 0.05). KEGG, Kyoto Encyclopedia of Genes and Genomes; DEG, differently expressed genes.

Seventeen protein-coding differently expressed genes (DEGs) and two lncRNAs (DE lncRNAs) were revealed through the RNA-Seq analysis (Log2FC ≥ 1, Log2FC ≤ −1, CPM ≥ 1, FDR < 0.05) in treated HCT116 cells when compared with the control group. We detected the upregulation of eight genes (*CYP2U1, NR1D1, ZNF804A, SEMA3C, DBP, NT5E, NAV3*, and *KDM5D*) and one lncRNA (LINC02043), as well as the downregulation of nine genes (*MUC16*, *KRT6A*, *ACVRL1*, *PTP4A1*, *HYAL1*, *CLIC3*, *TEX19*, *DES*, and *KCNK3*) and one lncRNAs (AL355075.4) (Figure 3A).

Additionally, we performed KEGG pathway enrichment analysis using all DEGs with FDR < 0.05. Three significantly enriched pathways for downregulated genes and one pathway for upregulated genes were identified (Figure 3B). These included “Wnt signaling pathway,” “TGF-beta signaling pathway,” “signaling pathways regulating pluripotency of stem cells,” and “circadian rhythm.”

### *NETO2* Gene Ortholog in Fish *N. furzeri*

**FIGURE 4:**
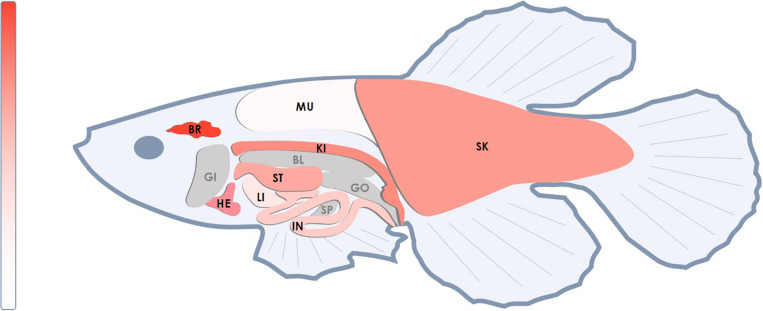
Relative mRNA levels of the *neto2b* gene in different tissues of *N. furzeri*. The highest mRNA level of *neto2b* was detected in the brain tissue and was taken as 1. The expression of *neto2b* in other studied tissues was measured relatively this value. Darker red indicates higher expression. Gray color indicates tissues where the gene expression has not been measured. BR, brain; HE, heart; ST, stomach; SK, skin; IN, intestines; LI, liver; KI, head kidney; MU, muscles; SP, spleen; BL, bladder; GI, gills; GO, gonads.

We analyzed the *NETO2* ortholog in the short-lived fish *N. furzeri*, termed *neto2b* (NCBI Gene ID: 107381994, location sgr07). Alignment of cDNA sequences demonstrated 69% of identity, whereas amino acid sequence alignment revealed higher similarity with 85% of conservative substitutions (positives) and only 2% of gaps (Supplementary Figure S1). In comparison, 71% of nucleotide sequences (cDNA) and 74% of amino acid sequence (6% of gaps) similarities were observed in *Danio rerio* (Supplementary Figure S2). Using qPCR, we measured *neto2b* expression in different tissues of *N. furzeri* (female) to estimate its potential use as a fish model presenting gene tissue-specific overexpression/downregulation. As observed in humans, *neto2b* demonstrated the highest mRNA levels in the fish brain. The expression of *neto2b* in other investigated tissues is presented in Figure 4.

## Discussion

A series of studies aimed at investigating the biological functions of *NETO2* has reported its crucial role in neural glutamate signaling. However, *NETO2* was also found to be upregulated in several tumors. Our research group was one of the first to reveal *NETO2* overexpression in solid tumors (renal and lung cancer) (Oparina et al., 2012). Recently, it has been reported that elevated *NETO2* expression was associated with tumor progression, poor prognosis, and reduced survival in cancer patients (Hu et al., 2015; He et al., 2019). In this study, we first demonstrated the deregulation of *NETO2* expression in breast and prostate cancer. The *NETO2* mRNA level was predominantly downregulated in breast cancer (44%), with elevated gene expression observed in 31% of samples. In prostate cancer, *NETO2* mRNA levels were upregulated in 40% of cases, with a 1.8 median change. Negligible correlations were observed between the *NETO2* expression and stage of breast and prostate cancer, as well as differentiation of prostate tumors. For colorectal cancer, we expanded a set of samples from a previous study (Fedorova et al., 2017) and confirmed that *NETO2* expression was increased in about one-third of these tumors (41 and 35% in previous and current studies, respectively). Nevertheless, a study evaluating Chinese patients with colorectal cancer has revealed the upregulation of *NETO2* in 52.6% of cases, reporting an association between expression and advanced tumor stage and invasion, poor differentiation, lymph node metastasis, and unfavorable prognosis in patients (Hu et al., 2015). In contrast, we failed to observe any significant correlations between the *NETO2* mRNA level and tumor stage, as well as differentiation that can be explained by the difference of the studied cohorts. Also, no association of *NETO2* expression with *KRAS*, *NRAS*, and *BRAF* mutations or MSI status were found.

Despite several studies indicating the involvement of *NETO2* in tumorigenesis, the underlying mechanism remains unclear. Li et al. (2019) have investigated *NETO2* functions in pancreatic cancer, and demonstrated that *NETO2* knockdown reduced the proliferative capacity of pancreatic cancer cells and suppressed tumor growth *in vivo*; *NETO2* overexpression conversely stimulated cell proliferation, invasion, and migration via the activation of the STAT3 pathway. Similar observations were reportedly documented following *NETO2* knockdown in nasopharyngeal carcinoma cells (He et al., 2019). The depletion of *NETO2* expression results in decreased proliferation, invasion, and migration of tumor cells and induced apoptosis via activating Caspase-3 signaling. Additionally, *NETO2* overexpression promotes the invasion and metastasis of gastric cancer cells by inducing epithelial-mesenchymal transition (EMT) by upregulating TNFRSF12A, which mediates the activation of the PI3K/AKT/NF-κB/Snail axis (Liu et al., 2019). In the present work, we demonstrated that stable *NETO2* knockdown in HCT116 cells resulted in the downregulation of Wnt and TGF-β signaling pathways, as well as signaling pathways regulating the pluripotency of stem cells. In the KEGG database, the “signaling pathways regulating pluripotency of stem cells” group comprises the Jak-STAT, MAPK, PI3K-Akt, Wnt, and TGF-β signaling pathways. All these stem cell-related pathways are extensively implicated in tumorigenesis (Dreesen and Brivanlou, 2007). Thus, our results demonstrated the participation of NETO2 in the deregulation of cell signaling in tumors and confirmed its relation to STAT and PI3K-Akt signaling as previously reported. Conversely, *NETO2* reduction leads to an upregulation of the circadian rhythm pathway in colorectal cancer cells. Circadian rhythm is closely associated with the cell cycle and has been implicated in DNA-damage response (Hunt and Sassone-Corsi, 2007), with the disruption of this pathway considered a risk factor for carcinogenesis (Straif et al., 2007). According to literature, NETO2 is related to cell proliferation and apoptosis in tumor cells (He et al., 2019; Li et al., 2019). These processes could be connection points between the functions of NETO2 and the circadian rhythm pathway.

Furthermore, we determined 17 genes presenting more than a 2-fold change in expression following *NETO2* knockdown. Increased expression was observed in genes involved in cell metabolism [*CYP2U1* (arachidonic acid metabolism) and *NT5E* (metabolism of nucleotides)], circadian rhythm (*NR1D1* and *DBP*), regulation of developmental processes (*SEMA3C* and *NAV3*), histone demethylation (*KDM5D*), and transcriptional regulation (*ZNF804A*). Decreased mRNA levels were noted for genes related to cell adhesion (*MUC16*), differentiation (*TEX19*), proliferation, and migration (*ACVRL1*, *PTP4A1*, and *HYAL1*), as well as cytoskeleton organization (*KRT6A* and *DES*) and ion transmembrane transport (*CLIC3* and *KCNK3*). Most genes (*CYP2U1*, *SEMA3C*, *NT5E*, *KRT6A*, *NAV3*, *ACVRL1*, *KCNK3*, *MUC16*, *PTP4A1*, and *HYAL1*) were previously found to be implicated in the development of colorectal cancer. Interestingly, *NETO2* is reportedly associated with the neuron navigator 3 (*NAV3*) gene, which is also predominantly expressed in the nervous tissue. Copy number changes in the *NAV3* gene have been observed in colorectal cancer (Carlsson et al., 2012). Moreover, *NAV3* is reportedly involved in the p73-mediated tumor suppression, as well as in Jak-STAT and GnRH signaling in colorectal cancer cells (Carlsson et al., 2012; Uboveja et al., 2020). Furthermore, the participation of *NETO2* has been revealed in the Jak-STAT signaling pathway during cancer. Additionally, *NETO2* silencing resulted in the altered expression of two lncRNAs, LINC02043, and AL355075.4, for which the functions and related cellular pathways have not been established. However, lncRNA LINC02043 was previously reported as a prognostic marker of recurrence-free survival in hepatocellular carcinoma (Luo et al., 2020). Notably, regulation of *NETO2* expression with microRNAs and microRNA-lncRNA interaction has been recently shown in esophageal cancer. *NETO2* was determined as a target of miR-206 and miR-143-5p, and participation of *NETO2* tumor progression and angiogenesis through overexpression of lncRNA FAM225A absorbed miR-206 was revealed (Wada et al., 2020; Zhang et al., 2020). LncRNAs found in our study can also be potential regulators of *NETO2* expression in colorectal cancer.

Although a wide range of studies has proposed increased *NETO2* expression in several tumors, we revealed its deregulation in breast, prostate, and colorectal cancers (both decreased and increased mRNA levels). The function of *NETO2* in tumorigenesis remains unclear; however, our findings and those obtained in previous studies indicate its participation in cancer-related signaling pathways. Moreover, all identified cancer-related signaling pathways (Wnt, TGF-β, STAT, MAPK, and PI3K-Akt) have previously been shown to be associated with breast, prostate, and colorectal cancer (Dhillon et al., 2007; Massagué, 2008; Pencik et al., 2016; Zhan et al., 2016; Jiang et al., 2020).

Finally, we analyzed the ortholog of the human *NETO2* gene in the unique animal model, the short-lived fish *N. furzeri*. This fish is characterized by an extremely short captive lifespan of 3 months. The use of *N. furzeri* for genetic studies allows for the rapid generation of transgenic lines and short-term experiments. We showed that the ortholog *neto2b* had the high similarity with human *NETO2* in nucleotide (69%) and amino acid (85%) sequences (even more than those for *D. rerio*), as well as stable expression in the majority of the fish tissues. These results confirm that *NETO2* is a very conservative gene among vertebrates and indicate the possibility to use *N. furzeri* for the study of the gene function and its role in tumorigenesis at the organism level.

## Data Availability Statement

The datasets generated for this study can be found in the online repositories. The names of the repository/repositories and accession number(s) can be found below: https://www.ncbi.nlm.nih.gov/sra/PRJNA655834.

## Ethics Statement

The studies involving human participants were reviewed and approved by the Ethics Committee of P.A. Hertsen Moscow Cancer Research Institute, Ministry of Health of the Russian Federation. The patients/participants provided their written informed consent to participate in this study. The animal study was reviewed and approved by the Bioethics Commission from the A. N. Severtsov Institute of Ecology and Evolution, Russian Academy of Sciences.

## Author Contributions

AS and AVK conceived and designed the work. NV clinically characterized tumor tissue samples. AL designed and maintained knockout cell line. MF, VP, and AAK performed the qPCR experiments. ZG, EP, MF, II, and MS performed the RNA isolation, library preparation, and western blot analysis. GK, DT, and VP carried out bioinformatics analysis. TD performed experimental procedures with the fish. AS, GS, and AVK wrote the manuscript. All authors read and approved the final manuscript.

## Conflict of Interest

The authors declare that the research was conducted in the absence of any commercial or financial relationships that could be construed as a potential conflict of interest.

## Footnotes

1 https://bgee.org/
