# Supplementary material for: NETO2 Is Deregulated in Breast, Prostate, and Colorectal Cancer and Participates in Cellular Signaling
Source: Front Genet. 2020 Dec 10;11:594933. doi: 10.3389/fgene.2020.594933 (PMC7758476; doi:10.3389/fgene.2020.594933)
Supplement: Supplementary file 1 [file Data_Sheet_1.doc]

**Table S1.** Clinicopathological characteristics of patients presenting colorectal cancer.

| Characteristics | Number, n |
| --- | --- |
| **Total number** | 74 |
| **Median age (range)**  ≥60  <60 | 23  51 |
| **Sex**  Male  Female | 28  46 |
| **Pathological stage**  I  II  III  IV | 9  20  32  13 |
| **Differentiation**  Well differentiated  Moderately differentiated  Poorly differentiated  Undifferentiated | 2  26  30  16 |
| **Primary tumor site**  Ascending colon  Cecum  Rectum  Sigmoid colon | 27  5  22  20 |
| **Metastasis**  Lymph node  Distance  Negative | 40  13  29 |
| ***KRAS* status**  Wild type  Mutated | 44  30 |
| ***NRAS* status**  Wild type  Mutated | 70  4 |
| ***BRAF* status**  Wild type  Mutated | 66  8 |
| **MSI status**  MSS + MSI-low  MSI-high | 61  13 |

CIMP, CpG island methylator phenotype; MSI, microsatellite instability; MSS, microsatellite stability.

**Table S2.** Clinicopathological characteristics of patients presenting prostate cancer.

| Characteristics | Number, n |
| --- | --- |
| **Total number** | 40 |
| **Median age (range)**  ≥60  <60 | 30  10 |
| **Pathological stage**  II  III | 30  10 |
| **Gleason score/Differentiation**  2-6/Well differentiated  7/Moderately differentiated  8-10/Poorly differentiated | 6  22  12 |
| **Prostate specific antigen (PSA), ng/ml**  ≤20  >20 | 31  9 |
| **Lymph node metastasis**  Positive  Negative | 0  40 |

**Table S3.** Clinicopathological characteristics of patients presenting breast cancer.

| Characteristics | Number, n |
| --- | --- |
| **Total number** | 32 |
| **Median age (range)**  ≥60  <60 | 15  17 |
| **Pathological stage**  I  II  III | 8  19  5 |
| **Differentiation**  Well differentiated  Moderately differentiated  Poorly differentiated | 1  30  1 |
| **Lymph node metastasis**  Positive  Negative | 20  12 |
| **Histological type**  Invasive lobular breast carcinoma  Invasive ductal breast carcinoma  Mixed ductal/lobular breast carcinoma  Tubular carcinoma | 3  27  1  1 |

**Table S4**. Sequences of primers and probes for reference genes.

| Gene | Oligo type | Sequence (5'-3') |
| --- | --- | --- |
| *GAPDH* | Forward primer  Reverse primer  Hydrolysis probe | GGAGTCAACGGATTTGGTC  TGGGTGGAATCATATTGGAACAT  CCTTCATTGACCTCAACTACATGGTTTACAT |
| *B2M* | Forward primer  Reverse primer  Hydrolysis probe | ATGAGTATGCCTGCCGTGTG  AATTCATCCAATCCAAATGCG  ATCTTCAAACCTCCATGATGCTGCTTACAT |
| *GUSB* | Forward primer  Reverse primer  Hydrolysis probe | GATGGAAGAAGTGGTGCGTAGG  TTAGAGTTGCTCACAAAGGTCACAG  CGTCCCACCTAGAATCTGCTGGCTACTACTT |

| **Score** | **Expect** | **Method** | **Identities** | **Positives** | **Gaps** |
| --- | --- | --- | --- | --- | --- |
| 807 bits (2084) | 0.0 | Compositional matrix adjust. | 376/517 (73%) | 443/517 (85%) | 15/517 (2%) |
|  |  |  |  |  |  |
| Query 3 RAWILFFLIEEGLALAQRTKDSLSESGIQSTNNQNDCGTWVRNINGGVFMSPNYPNTYPP | | | | | |
| + ++ L+ EG+A+AQ+T+D GI+ CG WVR NGG F SPNYP++YPP | | | | | |
| Sbjct 11 KVLLITVLVVEGIAVAQKTQDG-QNIGIKHIP-ATQCGIWVRTSNGGHFASPNYPDSYPP | | | | | |
|  |  |  |  |  |  |
| Query 63 NKECVYILEALPRQRIQLAFDKNYYIEPSFECRFDHIEIRDGPFGFSPLIDRFCGGKNPG | | | | | |
| NKEC+YILEA PRQRI+L FD++YYIEPSFECRFDH+E+RDGPFGFSPLIDR+CG K+P | | | | | |
| Sbjct 69 NKECIYILEAAPRQRIELTFDEHYYIEPSFECRFDHLEVRDGPFGFSPLIDRYCGVKSPP | | | | | |
|  |  |  |  |  |  |
| Query 123 LVTSTGRFMWIKFTSDEELEGLGFRIKYTFVADPDFHLHVGGLLNPIPDCQFEVGGWDGI | | | | | |
| L+ STGRFMWIKF+SDEELEGLGFR KY+F+ DPDF ++GG+LNPIPDCQFE+ G DGI | | | | | |
| Sbjct 129 LIRSTGRFMWIKFSSDEELEGLGFRAKYSFIPDPDF-TYLGGILNPIPDCQFELSGADGI | | | | | |
|  |  |  |  |  |  |
| Query 183 IRSSQVEEEDRVKPGDALDCIWTIKAPPKSKIYLRFIDYQMEHSNECKKNFVAVYDGSSA | | | | | |
| +RSSQVE+E++ KPG A+DCIWTIKA PK+KIYLRF+DYQMEHSNECK+NFVAVYDGSS+ | | | | | |
| Sbjct 188 VRSSQVEQEEKTKPGQAVDCIWTIKATPKAKIYLRFLDYQMEHSNECKRNFVAVYDGSSS | | | | | |
|  |  |  |  |  |  |
| Query 243 IENLKAKFCSTVANDLMLDNGVGVVRMWADEKSRLSRFRMLFTSYVDPPCTASTFFCHSN | | | | | |
| IENLKAKFCSTVAND+ML G+GV+RMWADE SRLSRFRMLFTS+V+PPCT+STFFCHSN | | | | | |
| Sbjct 248 IENLKAKFCSTVANDVMLKTGIGVIRMWADEGSRLSRFRMLFTSFVEPPCTSSTFFCHSN | | | | | |
|  |  |  |  |  |  |
| Query 303 MCINNSLVCNGIQNCVYPWDENHCKETQSKGLFHQITKTHGTVIGVSSGIVLVLLIISIL | | | | | |
| MCINNSLVCNG+QNC YPWDENHCKE + G+F QITKTHGT+IG++SGIVLVLLIISIL | | | | | |
| Sbjct 308 MCINNSLVCNGVQNCAYPWDENHCKEKKKAGVFEQITKTHGTIIGITSGIVLVLLIISIL | | | | | |
|  |  |  |  |  |  |
| Query 363 VQMKQPRKKVVARRPGVFNKAGFQEVFDPPHYELFSLRDKEMSSDLADLSEELDSFHKLR | | | | | |
| VQ+KQPRKKV+A + FNK GFQEVFDPPHYELFSLRDKE+S+DLADLSEELD++ K+R | | | | | |
| Sbjct 368 VQVKQPRKKVMACKTA-FNKTGFQEVFDPPHYELFSLRDKEISADLADLSEELDNYQKMR | | | | | |
|  |  |  |  |  |  |
| Query 423 RSSTMSRCVHEHHCGSQASVATGGGSMKHSRTTLSSMELSYHNDFSKPPPMKTFNSTSTY | | | | | |
| RSST SRC+H+HHCGSQAS S+K SRT LSSMEL + NDF++P PMKTFN ST+ | | | | | |
| Sbjct 427 RSSTASRCIHDHHCGSQAS------SVKQSRTNLSSMELPFRNDFAQPQPMKTFN--STF | | | | | |
|  |  |  |  |  |  |
| Query 483 KKSCYGYKQHAQTHDCDQQVIEDRVAEEIPCEIYGRG 519 | | | | | |
| KKS Y +K Q H+C +Q +EDRV EEIPCEIY RG | | | | | |
| Sbjct 479 KKSSYTFK---QGHECPEQALEDRVMEEIPCEIYVRG 512 | | | | | |

**Figure S1.** Protein sequence alignment of the human *NETO2* gene with *N. furzeri*. Query – fish protein sequence, Sbjct – human protein sequence.

|  | | | | | |  |  |  |  |  |
| --- | --- | --- | --- | --- | --- | --- | --- | --- | --- | --- |
| **Score** | **Expect** | **Method** | **Identities** | **Positives** | **Gaps** |  |  |  |  |  |
| 634 bits (1636) | 0.0 | Compositional matrix adjust. | 300/541 (55%) | 402/541 (74%) | 36/541 (6%) |  |  |  |  |  |
|  |  |  |  |  |  |  |  |  |  |  |
| Query 2 LVLLILIEEGFALAQKTQVALAQNVAVADKKPAHCGTLVQTENGGTFSSPNYPSTYPPNK 61 | | | | | | | | | | |
| L++ +L+ EG A+AQKTQ QN+ + CG V+T NGG F+SPNYP +YPPNK | | | | | | | | | | |
| Sbjct 13 LLITVLVVEGIAVAQKTQDG--QNIGIKHIPATQCGIWVRTSNGGHFASPNYPDSYPPNK 70 | | | | | | | | | | |
|  |  |  |  |  |  |  |  |  |  |  |
| Query 62 ECVYILEAHPRKRIQLVFDDIYHIEPSFECRFDNIEIRDGPFIFSPLINRFCGDKSPGIV 121 | | | | | | | | | | |
| EC+YILEA PR+RI+L FD+ Y+IEPSFECRFD++E+RDGPF FSPLI+R+CG KSP ++ | | | | | | | | | | |
| Sbjct 71 ECIYILEAAPRQRIELTFDEHYYIEPSFECRFDHLEVRDGPFGFSPLIDRYCGVKSPPLI 130 | | | | | | | | | | |
|  |  |  |  |  |  |  |  |  |  |  |
| Query 122 TSSGRFLWIKFTSDEELEELGFKVEYSYTADPDFHLHVGGLLNPIPDCQFEMSGADGVIR 181 | | | | | | | | | | |
| S+GRF+WIKF+SDEELE LGF+ +YS+ DPDF ++GG+LNPIPDCQFE+SGADG++R | | | | | | | | | | |
| Sbjct 131 RSTGRFMWIKFSSDEELEGLGFRAKYSFIPDPDF-TYLGGILNPIPDCQFELSGADGIVR 189 | | | | | | | | | | |
|  |  |  |  |  |  |  |  |  |  |  |
| Query 182 SSQVEEENKVKAGEAVDCIWTIRAPPMSKIYLRFLDYQLENSNECKKNFVAVYEGSNAIE 241 | | | | | | | | | | |
| SSQVE+E K K G+AVDCIWTI+A P +KIYLRFLDYQ+E+SNECK+NFVAVY+GS++IE | | | | | | | | | | |
| Sbjct 190 SSQVEQEEKTKPGQAVDCIWTIKATPKAKIYLRFLDYQMEHSNECKRNFVAVYDGSSSIE 249 | | | | | | | | | | |
|  |  |  |  |  |  |  |  |  |  |  |
| Query 242 DLKAKFCSTVANDITLDNTVAVVRMWADETSKLSRFRLLFTVFTEPPCSPNAYFCHSNMC 301 | | | | | | | | | | |
| +LKAKFCSTVAND+ L + V+RMWADE S+LSRFR+LFT F EPPC+ + +FCHSNMC | | | | | | | | | | |
| Sbjct 250 NLKAKFCSTVANDVMLKTGIGVIRMWADEGSRLSRFRMLFTSFVEPPCTSSTFFCHSNMC 309 | | | | | | | | | | |
|  |  |  |  |  |  |  |  |  |  |  |
| Query 302 INNTLVCNGVQNCVFPWDENNCREKKPKTFFHQMSKTHGTVIGVSTGVVCLLLIISIFIQ 361 | | | | | | | | | | |
| INN+LVCNGVQNC +PWDEN+C+EKK F Q++KTHGT+IG+++G+V +LLIISI +Q | | | | | | | | | | |
| Sbjct 310 INNSLVCNGVQNCAYPWDENHCKEKKKAGVFEQITKTHGTIIGITSGIVLVLLIISILVQ 369 | | | | | | | | | | |
|  |  |  |  |  |  |  |  |  |  |  |
| Query 362 MKQPRKKILCRKGLFSATEMQEVMDPPEYEMFSMQDTDMPEEL--LEEELEGLQKLHRSA 419 | | | | | | | | | | |
| +KQPRKK++ K F+ T QEV DPP YE+FS++D ++ +L L EEL+ QK+ RS+ | | | | | | | | | | |
| Sbjct 370 VKQPRKKVMACKTAFNKTGFQEVFDPPHYELFSLRDKEISADLADLSEELDNYQKMRRSS 429 | | | | | | | | | | |
|  |  |  |  |  |  |  |  |  |  |  |
| Query 420 SMSRCIREHHCGAHNSTTILMTRTN------PLSEELSTAVGARSWGSFHGRRSSSRSHH 473 | | | | | | | | | | |
| + SRCI +HHCG+ ++++ +RTN P + + +++ S | | | | | | | | | | |
| Sbjct 430 TASRCIHDHHCGSQ-ASSVKQSRTNLSSMELPFRNDFAQPQPMKTFNS------------ 476 | | | | | | | | | | |
|  |  |  |  |  |  |  |  |  |  |  |
| Query 474 PHTHAYSAQSLREALEEDELSLEGQVMEEIGYNEVFGRGRGVVMVRNHTNPVQQRSLSMD 533 | | | | | | | | | | |
| T S+ + ++ E E +LE +VMEEI E++ RGR Q S+S+D | | | | | | | | | | |
| Sbjct 477 --TFKKSSYTFKQGHECPEQALEDRVMEEIPC-EIYVRGR---------EDSAQASISID 524 | | | | | | | | | | |
|  |  |  |  |  |  |  |  |  |  |  |
| Query 534 F 534 | | |  |  |  |  |  |  |  |  |
| F | |  |  |  |  |  |  |  |  |  |
| Sbjct 525 F 525 | | |  |  |  |  |  |  |  |  |

**Figure S2.** Protein sequence alignment of the human *NETO2* gene with *D. rerio*. Query – fish protein sequence, Sbjct – human protein sequence.
